# Use and misuse of prescription stimulants by university students: a cross-sectional survey in the french-speaking community of Belgium, 2018

**DOI:** 10.1186/s13690-022-00816-3

**Published:** 2022-02-16

**Authors:** Martine Sabbe, Javier Sawchik, Mégane Gräfe, Françoise Wuillaume, Sara De Bruyn, Pierre Van Antwerpen, Guido Van Hal, Martin Desseilles, Jamila Hamdani, Hugues Malonne

**Affiliations:** 1 Scientific Directorate of Epidemiology and public health – Sciensano, Brussels, Belgium; 2 DG Post authorisation - Federal Agency for Medicines and Health Products, Brussels, Belgium; 3 Unit of Pharmacology, Pharmacotherapy and Pharmaceutical Care, Faculty of Pharmacy, Université libre de Bruxelles, Brussels, Belgium; 4 Department of Sociology, University of Antwerp, Antwerp, Belgium; 5 RD3-Pharmacognosy, Bioanalysis and Drug Discovery- Faculty of Pharmacy, Université libre de Bruxelles, Brussels, Belgium; 6 Unit of Epidemiology and Social Medicine, University of Antwerp, Antwerp, Belgium; 7 Faculty of Medicine, Université de Namur, Namur, Belgium; 8 Department of Biomedical Sciences, Namur Research Institute for Life Sciences, University of Namur, Namur, Belgium

**Keywords:** Misuse of prescription drugs, Belgium, Stimulants, Drug misuse, Methylphenidate, Survey, University

## Abstract

**Background:**

Misuse of prescription stimulants (PS) has been reported among students to enhance academic performance in Flanders (Belgium). However, PS misuse among students in the French-speaking community is unknown. The main purpose of the study was to estimate the prevalence of medical use and misuse of PS by university students in the French-speaking community (Belgium), and to investigate the reasons and sources associated with PS misuse.

**Methods:**

A cross-sectional online survey was performed in 2018. All university students 18 years and older were invited to participate and asked about PS use, including medical (i.e., used for therapeutic purposes) and nonmedical reasons and sources of PS.

**Results:**

In total, 12 144 students participated in the survey (median age = 21 years, 65.5% female). The estimated prevalence of PS use was 6.9% (ever use) and 5.5% (past-year). Among ever users, 34.7% were classified as medical users and 65.3% as misusers. Lifetime prevalence of misuse was estimated at 4.5%. The most common reason for medical use was treatment of attention disorder (85.9%). Reasons for misuse were mainly to improve concentration (76.1%) or to stay awake and study longer (50.7%). Friends or acquaintances inside the student community and general practitioners were the main sources of PS for misuse (41.5% and 23.5%, respectively).

**Conclusions:**

This study found that rates of misuse of PS in French-speaking universities in Belgium were in line with studies conducted in Flanders and Europe. Academic institutions can use these results to tailor their drug prevention campaigns.

## Background

Stimulants (e.g. methylphenidate [MPH]) and stimulant-like medications (e.g. modafinil), hereinafter referred to as prescription stimulants (PS), are indicated to treat attention-deficit/hyperactivity disorder (ADHD) and narcolepsy. The use of these drugs in adults has been a matter of debate, especially since the publication and subsequent withdrawal of a Cochrane systematic review on immediate-release MPH for adults with ADHD [1]. Moreover, questions have raised on the increased misuse by healthy students for neuroenhancement, i.e. to increase cognition, vigilance, motivation and productivity [2, 3]. According to the Global Drug Survey, an increase of neuroenhancement with prescription and illegal stimulants was noted across all surveyed countries [4]. However, in this study self-selected samples from cross-sectional studies are compared and samples should not be considered representative of the country general population. According to a systematic review, self-reported rates of non-medical use of prescription stimulants ranged from 2 to 59% in the US and Canada [5]. According to the European Monitoring Centre for Drugs and Drug Addiction, work to better understand the extent and nature of non-medical use of medicines and monitor developments is underway in Europe [6]. In a review in 2015, most European studies reported lower rates of misuse of prescription drugs for neuroenhancement compared to the US [2]. In Germany, the lifetime prevalence of PS misuse by students was 4.6% in 2010, while prevalence was 11% in Italy in 2014-2015 and 6.2% in Switzerland in 2011 [7–9].

Misuse and abuse of PS can be of concern particularly in ADHD patients with diagnoses of conduct or substance use disorder [10]. While evidence of abuse or dependence on ADHD drugs is rare, misuse and diversion of these medications in individuals not diagnosed with ADHD is frequently reported [5, 11, 12]. Adverse effects associated to misuse of MPH can be related to the cardiovascular system (e.g. arrhythmia, hypertension), the central nervous system (e.g. aggressiveness, confusion, headache, mood swings) and the gastrointestinal system (e.g. abdominal pain, anorexia) [13, 14]. Of note is that in MPH-naive subjects, a toxic dose may be very close to the therapeutic dose when compared with patients under long-term treatment [13].

In Belgium, the consumption of MPH increased from 4.5 million Defined Daily Dose (DDD) in 2004 to 10.3 million DDD in 2011 [15]. This increase can probably be explained by a greater awareness and attention for ADHD. However, there is no information about the number of diagnoses in children or adults that have been made. Development of clinical, preventive, political, and educational strategies for reducing PS misuse requires knowledge of the extent to which PS are used, reasons for their use and sources of acquisition. A survey conducted among university and university college students in Flanders estimated the lifetime prevalence of use of PS at 11% in 2017 [16]. In addition, 8.5% of these students have used PS to improve their study performance without being part of a treatment. A study among medical students in Flanders indicated that 8.7% of the respondents used PS during the exam period to enhance their study performances [17]. However, prevalence of use and misuse of PS in Belgian French-speaking universities is unknown.

## Methods

### Aim of the study

The main objective of this study was to study the prevalence of medical use and misuse of PS among students in French-speaking Belgian universities. The study also sought to better understand the reasons for misuse, as well as to assess perceived effects, sources of PS acquisition and association with the use of other substances.

### Study design and setting

A cross-sectional study was performed using an online survey in the six French-speaking universities in Belgium (Université libre de Bruxelles, Université catholique de Louvain, Université de Liège, Université de Mons, Université de Namur and Université Saint-Louis – Bruxelles). All students, aged 18 year or over, were invited to participate via an email sent by the academic authorities. Student organizations, such as the Inter-University Committee for Medical Students, reinforced the invitations through e-mails or social networks.

This study was approved by the ethical committee of the Erasme University Hospital (ULB) and was conducted between October and November 2018 (Reference: P2018/447). Different definitions and classifications are used throughout literature for misuse; here we classified the type of use of PS as medical when PS were used for therapeutic purposes and as misuse when PS were not part of a treatment.

### Survey tool

Based on previous studies, a questionnaire was developed in French language [17, 18]. Thirty-seven questions spread over 37 pages were displayed using skip patterns where necessary. The first section of the questionnaire was designed to collect data about use of ADHD and narcolepsy medication. The following drugs, marketed in Belgium at the time of the survey, were listed : MPH, modafinil, atomoxetine, guanfacine, and sodium oxybate. The latter three drugs, although not considered stimulants, can also be potentially misused for similar purposes as PS. An additional free-text option was presented for other possible medications. Since this article focuses on the use and misuse of PS, we selected MPH and modafinil (referred to as PS in this article) for further analysis because these are psychostimulants or have stimulant-like effects. Additional questions collected data on sociodemographic variables (gender, age, study year, working and housing status) and use of other substances (such as alcohol, nicotine, energy drinks, hypnotics, sedatives, corticosteroids, cannabis, amphetamines, ecstasy or cocaine). In addition, age of first use of PS (6-11 years, 12-17 years, ≥18 years) and frequency of use in the last month were questioned. For reasons of use, source and perceived effects, multiple answers were possible. The order of response choices was randomized for each respondent. The time required to complete the questionnaire was estimated at less than 15 min. The questionnaire was preceded by a cover letter inviting students to participate, stating that participation was voluntary, guaranteeing the confidentiality and anonymity of the data, and describing the main purpose of the survey.

SurveyMonkey was used to host the questionnaire and to collect responses [19]. Personal data and IP addresses were not collected. Informed consent was given by voluntary completion of the questionnaire. Multiple responses from the same device were not allowed. Response editing was allowed, so respondents could modify their answers until they had completed the questionnaire. Two reminders were implemented to increase the potential response rate. No financial incentives were proposed.

### Sample size

The total number of students enrolled in Belgian French-speaking universities was 88 783 for the academic year 2013-2014; this figure was used as the reference population as this was the most recent one available at the time of the survey [20]. A recent survey in Flanders obtained a response rate of 14%, with considerable variation between the institutions [16]. We have conservatively assumed that the expected response rate would be about half that obtained in the above-mentioned survey (between 5% and 7%). It was therefore expected that the number of respondents would be between 4 500 and 6 000. Assuming a prevalence of use of 10% (based on the survey in Flanders in 2017 [16]), the margin of error is about 0.6% points: this was considered as an appropriate level of precision.

### Data analysis

The response rate was calculated by dividing the number of respondents (subjects who answered to the first question) by the reference population. The completeness rate was calculated by dividing the number of respondents who answered all questions except the last open-ended question by the total number of respondents.

The sociodemographic characteristics of the study population were summarized, overall and by type of use. The age distribution was described using descriptive statistics, while categorical variables were summarized with frequencies and proportions (expressed as percentages). The 95% confidence intervals (95% CI) associated with the proportions were calculated using the Clopper-Pearson method.

Lifetime prevalence of use was estimated as the proportion of the study population who responded to have used PS at some point in time, and was calculated overall, by type of use and specific drug. In addition, the past-year prevalence of use (i.e., in the past 12 months) was also estimated. To better characterize the profile of misusers, proportions were calculated by age of onset (6-11 years, 12-17 years, ≥18 years), reason, source, and perceived effects. All the responses (from fully and partially completed questionnaires) were included in the data analysis. Partial respondents were not included in the calculations of the proportions of unanswered questions. Missing values were not imputed.

Potential associations between the use of PS and student characteristics were explored. Categorical variables were reclassified as follows: (1) gender: male or female (2) school year: 1st year bachelor or other year, (3) working status: yes (including vacation job) or no, (4) housing status: alone, flat sharing, with parents/family (5) field of study: medicine or other, and (6) use of commonly misused/abused substances: ≤1/month or >1/month. Age was modelled as a continuous variable. First, potential predictors of PS use and misuse were selected with the GLMSELECT SAS procedure (using stepwise selection with 5-fold cross-validation as choose criterion). A multinomial logistic regression model was then fitted to explore the relationships between the selected potential predictors and type of use of PS (non-use, medical use, misuse). Non-users was the category chosen as the reference. Multivariable odds ratios and associated 95% profile-likelihood CIs were calculated.

## Results

### Sample description

A total of 12 144 subjects responded to the survey. The response rate was estimated at approximately 14% (12 144/88 783). A total of 514 subjects were excluded from the analysis: 504 subjects older than 30 years, 9 younger than 18 years and 1 subject due to highly inconsistent and incomplete answers. The final analysis dataset consisted of 11 630 subjects. The completeness rate was estimated at 90% (10 518/11 630).

Socio-demographic characteristics of the respondents are summarized in Table 1. In the study population, 65.5% were females, 34.0% were males, and the median age was 21 years.

**Table 1:** Socio-demographic characteristics overall and by use of prescription stimulants, survey in French-speaking universities, Belgium, 2018

Characteristic	Users *N*=803	No users *N*=10 827	Overall *N*=11 630
Age (years) n Mean (SD) Median (Q1-Q3)	729 22.4 (2.9) 22 (20-24)	10 363 21.1 (2.8) 21 (19-23)	11,092 21.2 (2.8) 21 (19-23)
Gender n Female Male Other	732 360 (49.2, 45.6-52.8) 363 (49.6, 46.0-52.2) 9 (1.2, 0.6-2.3)	10 401 6 932 (66.6, 65.7-67.6) 3 420 (32.9, 32.0-33.8) 49 (0.5, 0.3-0.6)	11 133 7 292 (65.5, 64.6-66.3)) 3 783 (34.0, 33.1-34.9) 58 (0.5, 0.4-0.7)
School year n Bachelor (1st year) Bachelor (other) Master	715 160 (22.4, 19.4-25.6) 237 (33.1, 29.7-36.7) 318 (44.5, 40.8-48.2)	10 199 3 319 (32.5, 31.6-33.5) 3 139 (30.8, 29.9-31.7) 3 741 (36.7, 35.7-37.6)	10 914 3 479 (31.9, 31.0-32.8) 3 376 (30.9, 30.1-31.8) 4 059 (37.2, 36.3-38.1)
Working status No job Vacation job Job < 20 h/week Job ≥ 20 h/week	716 286 (39.9, 36.3-43.6) 144 (20.1, 17.2-23.2) 191 (26.7, 23.5-30.1) 95 (13.3, 10.9-16.0)	10 161 4 630 (45.6, 44.6-46.5) 2 718 (26.7, 25.9-27.6) 2 152 (21.2, 20.4-22.0) 661 (6.5, 6.0-7.0)	10 877 4 916 (45.2, 44.3-46.1) 2 862 (26.3, 25.5-27.2) 2 343 (21.5, 20.8-22.3) 756 (7.0, 6.5-7.4)
Housing status n With parents/family Flat sharing Alone Other	713 331 (46.4, 42.8-50.1) 214 (30.0, 26.7-33.6) 150 (21.0, 18.1-24.2) 18 (2.5, 1.5-4.0)	10 132 5 529 (51.6, 50.6-52.6) 3 007 (29.7, 28.8-60.6) 1 619 (16.0, 15.3-16.7) 277 (2.7, 2.4-3.1)	10 845 5 560 (51.3, 50.3-52.2) 3 221 (29.7, 28.8-30.6) 1 769 (16.3, 15.6-17.0) 295 (2.7, 2.4-3.0)

Descriptive statistics for age, n (%, 95%CI) for categorical variables

### Prevalence and reasons of (mis)use

A total of 803 subjects reported having used PS giving a lifetime prevalence of PS use of 6.9% (95% CI 6.5%-7.4%). Males were more likely to be PS users than females (9.6% vs. 4.9%). Past-year use was reported by 638 subjects giving a prevalence estimate of 5.5% (95%CI 5.1-5.9%).

Among PS users, 277 (34.7%, 95% CI 31.4%-38.1%) were classified as medical users and 522 (65.3%, 95%CI 62.0-68.6%) as misusers. The prevalence of lifetime misuse in the overall study population was 4.5% (95%CI 4.1-4.9%). Among medical users, 26 subjects (9.5%, 95%CI 6.2-13.6%) reported to have used PS also for other purposes. Of the PS users, 763 subjects (95.0%) and 81 subjects (10.1%) reported using MPH and modafinil, respectively. Other reported drugs were: atomoxetine (n=38), oxybate (n=34) and guanfacine (n=30). Sixty subjects reported use of other drugs, including 12 subjects who reported using amphetamines or dextroamphetamine.

The prevalence of PS use was higher among those who responded only partially to the questionnaire (13.8%, 95%CI 12.0-15.9%) compared to those who responded to all questions (6.1%, 95%CI 5.6-6.6%).

The majority of the medical users reported using PS for the treatment of attention disorder (n=238, 85.9%, 95% CI 81.3%-89.8%). Hyperactivity and narcolepsy were reported by 109 (39.3%, 95% CI 33.6%-45.1%) and 24 subjects (8.7%, 95% CI 5.6%-12.6%), respectively. Some other indications cited included autism, dyslexia, depression, epilepsy, hypersomnia, psychosis, post-traumatic stress disorder, anxiety disorders and sleep disorders. Among misusers, the most common reasons were to concentrate when studying and to stay awake and study longer (Table 2).

**Table 2:** Reasons for using prescription stimulants for purposes other than treatment, survey in French-speaking universities, Belgium, 2018

Reasons	n (%) (*N* = 544)	95% Clopper-Pearson CI
To concentrate when studying	414 (76.1)	72.3-79.7
To stay awake and study longer	276 (50.7)	46.5-55.0
To improve intellectual performance	256 (47.1)	42.9-51.3
To better memorize the courses	240 (44.1)	39.9-48.4
To try	116 (21.3)	18.0-25.0
To party	42 (7.7)	5.6-10.3
To disconnect from reality	27 (5.0)	3.3-7.1
To lose weight	27 (5.0)	3.3-7.1
To improve sport performance	18 (3.3)	2.0-5.2
To do like others	8 (1.5)	0.6-2.9
Other	36 (6.6)	4.7-9.0

Only 25 medical users reported reasons other than treatment for use of prescription stimulants

### Patterns of timing and age of first-use

Regular use (i.e., at least 1x/week) was common during the examination/revision period for both medical users (n=204, 82.0%) and misusers (n=274, 56.5%). Regular use during the year was more common for medical users (n=150, 59.1%), compared to misusers (n=37, 8.6%).

The majority of misusers started using PS from the age of 18 years or older (n=420, 82.5%), whereas the majority of medical users started before the age 18 (n=181, 66.5%).

### Sources and routes of administration

The vast majority of medical users rely on health care professionals. On the other hand, misusers obtained the drugs mainly from the student community or from a general practitioner (GP) (Table 3).

**Table 3:** Sources for obtaining prescription stimulants for medical use and misuse, survey in French-speaking universities, Belgium, 2018

Source	Medical users (*N*=277)		Misusers (*N*=522)		*p*-value^a^
	**n (%)**	**95%CI)**	**n (%)**	**95%CI**	
Peers (student community)	6 (2.3)	0.9-5.0	199 (41.5)	37.0-45.9	<0.0001
General practitioner	99 (38.1)	32.1-44.0	113 (23.5)	19.8-27.6	<0.0001
Friends / acquaintances	7 (2.7)	1.1-5.5	75 (15.6)	12.5-19.2	<0.0001
Sibling / other family member	6 (2.3)	0.9-5.0	57 (11.9)	9.1-15.1	<0.0001
Parents	9 (3.5)	1.6-6.5	50 (10.4)	7.8-13.5	0.0009
Internet	3 (1.2)	0.2-3.3	45 (9.4)	6.9-12.3	<0.0001
Neurologist / psychiatrist	220 (84.6)	79.6-88.8	39 (8.1)	5.8-10.9	<0.0001
Theft (drug/prescription)	3 (1.2)	0.2-3.3	7 (1.5)	0.6-3.0	1.00^b^
Other	6 (2.3)	0.9-5.0	19 (4.0)	2.4-6.1	0.2

95%CI: Clopper-Pearson confidence interval

^a^ Chi-square test, ^b^ Fisher’s exact test (25% of the cells have expected counts less than 5)

The oral route was by far the most frequently reported (*n*=742, 98.7%). Nasal administration was more commonly reported in misusers (*n*=52, 10.6%) compared to medical users (*n*=3, 1.1%). Inhalation (*n*=19, 2.5%) and injection (*n*=11, 1.5%) were reported rarely

### Perceived effects

A total of 425 subjects among 765 users (55.6%, 95% CI 52.0%-59.1%) reported having experienced adverse events (AEs) after using PS. Over half of the respondents reported having suffered from sleep disorders (*n*=245, 57.6%). Other commonly reported AEs were palpitations (*n*=201, 47.3%) and emotional instability (*n*=189, 44.5%).

A total of 650 subjects among 760 users (85.5%, 95%CI 82.8-88.0%) reported having experienced positive effects after using PS. The vast majority of the respondents reported an improvement in concentration (*n*=595, 91.5%). Other frequently reported positive effects were related to the ability to stay awake (*n*=317, 48.8%), motivation to study (*n*=320, 49.2%), increased energy (*n*=296, 45.5%) or improved academic performance (*n*=303, 46.6%). See Tables 4 and 5 for a comparison of perceived effects between misusers and medical users.

**Table 4:** Perceived negative effects associated with the use of prescription stimulants, survey in French-speaking universities, Belgium, 2018

Negative effects	Medical users (*N*=180)		Misusers (*N*=245)		*p*-value^b^
	**n** (%)	**95%CI**†	**n** (%)	**95%CI**^a^	
Sleep disorders	103 (57.2)	49.6-64.6	142 (58.0)	51.5-64.2	0.9
Palpitations	70 (38.9)	31.7-46.4	131 (53.8)	47.0-59.8	0.003
Emotional instability	84 (46.7)	39.2-54.2	105 (42.9)	36.6-49.3	0.4
Agitation	47 (26.1)	19.9-33.2	93 (38.0)	31.9-44.4	0.01
Aggressiveness	54 (30.0)	23.4-37.3	71 (29.0)	23.4-35.1	0.8
Headaches	33 (18.3)	13.0-24.8	73 (29.8)	24.1-35.9	0.007
Depression	62 (34.4)	27.5-41.9	57 (23.3)	18.1-29.1	0.01
Nausea	47 (26.1)	19.9-33.2	40 (16.3)	11.9-21.6	0.01
Anorexia	51 (28.3)	21.8-35.5	39 (15.9)	11.6-21.1	0.002
Suicidal thoughts	26 (14.4)	9.7-20.4	27 (11.0)	7.4-15.6	0.3
Hallucinations	9 (5.0)	2.3-9.3	12 (4.9)	2.6-8.4	>0.9
Other	33 (18.3)	13.0-24.8	28 (11.4)	7.7-16.1	0.04

^a^95%CI: Clopper-Pearson confidence interval

^b^Chi-square test

**Table 5:** Perceived positive effects associated with the use of prescription stimulants, survey in French-speaking universities, Belgium, 2018

Positive effects	Medical users (*N*=254)		Misusers (*N*=441)		*p*-value^b^
	**n** (%)	**95%CI**^a^	**n** (%)	**95%CI**†	
Improved concentration	231 (95.9)	92.5-98.0	364 (89.0)	85.6-91.9	0.002
Stay awake / study longer	82 (34.0)	28.1-40.4	235 (57.5)	52.5-62.3	<0.0001
Motivation to study	115 (47.7)	41.3-54.2	205 (50.1)	45.3-55.0	0.6
More energy	92 (38.2)	32.0-44.6	204 (49.9)	45.0-54.7	0.004
Improved results	133 (55.2)	48.7-61.6	170 (41.6)	36.7-46.5	0.0008
Sport performance	16 (6.6)	3.8-10.§	14 (3.4)	1.9-5.7	0.06
Other	44 (17.4)	12.3-22.6	46 (10.6)	7.8-13.8	0.01

^a^95%CI: Clopper-Pearson confidence interval

^b^Chi-square test

### Predictors of PS use

In general, the logistic regression models showed a similar pattern of associations between predictors and use of PS (for both medical use and misuse vs. non-use). Users of PS were more likely to be older, male, and to be users of other substances. (Table 6).

**Table 6:** Association between use of stimulants, sociodemographics and other substances, survey in French-speaking universities, Belgium, 2018

Covariate	Medical users (*N*=241) vs. No users (*N*=9 810)		Misusers (*N*=410) vs. No users (*N*=9 810)	
	**OR**	**% (95%CI)**	**OR**	**% (95%CI)**
Age (years)	1.11	1.06-1.16^c^	1.19	1.15-1.24^c^
Gender^a^	1.83	1.43-2.42^c^	1.87	1.52-2.32^c^
Nicotine^b^	1.48	1.04-2.07^c^	2.29	1.78-2.92^c^
Energy drinks^b^	1.11	0.81-1.50	2.22	1.78-2.76^c^
Hypnotics, Sedatives^b^	2.21	1.36-3.44^c^	2.04	1.42-2.89^c^
Corticosteroids^b^	4.48	2.25-8.22^c^	2.44	1.26-4.47^c^
Cannabis^b^	1.48	0.97-2.21	1.90	1.43-2.51^c^
Amphetamines^b^	3.35	0.83-11.08	2.13	0.84-5.39^c^
Ecstasy^b^	4.12	0.75-17.99	1.93	0.70-5.18
Cocaine^b^	0.28	0.03-1.55	4.51	2.08-9.51^c^

Odds Ratio (OR) and 95% Profile-likelihood confidence interval obtained from a multinomial logistic model

^a^male vs. female, ^b^>1month vs. ≤ 1 month, ^c^OR statistical significant at the 5% level

## Discussion

### Prevalence and reasons of (mis)use

This is the first survey estimating the prevalence of PS use among students at French-speaking universities in Belgium. We estimated a lifetime and past-year prevalence of use of 6.9% and 5.5%, respectively, and a lifetime prevalence of misuse of 4.5%. These results are in line with studies of Dutch-speaking university and college students, which reported in 2017 a lifetime prevalence of PS use of 10,5%, a past-year prevalence of 6.5% and a lifetime prevalence of misuse of 8.5% [16]. Previous studies conducted between 2005 and 2013 among college and university students in Flanders did not indicate an increase in PS use over time [21]. Our findings are also consistent with estimates from the UK and Ireland, where less than 10% of students reported a lifetime prevalence of PS use for neuroenhancement purposes [22].

We found that lifetime use was twice as high in males as in females and increased with age, confirming trends observed in other studies [2, 22, 23]. It should be noted, however, that there is no significant gender difference in the diagnosis of ADHD in adulthood, in contrast to childhood when the prevalence is higher in boys [24].

Among students using PS for medical reasons, attention deficit disorder was the most frequently cited indication. In our study, the primary reasons for PS misuse were academically related, which is consistent with a review study in which academic motivations were cited by 50–89% of students [5]. While in that study the second most commonly cited reason for non-medical use was “to get high”, we found that misuse as a party drug was rather limited. Furthermore, while there are cases of ADHD with apparent onset in adulthood, it is likely that many of these cases were not previously diagnosed [25]. It is therefore possible that some students are treating themselves with illicitly acquired PS. Thus, the desire to improve cognitive performance may be a form of self-treatment for undiagnosed ADHD [26]. ADHD is common in adults, with an estimated prevalence of 3.6% in high-income countries [27] and it is possible that a number of students have ADHD that is not being addressed and face barriers to receiving diagnostic evaluation and treatment. Nevertheless, although students may begin to misuse PS due to poor grades, misuse appears to be negatively associated with academic performance [28, 29].

Interestingly, one in ten medical users in our survey also reported use of their PS for purposes other than treatment. ADHD patients may use their PS in greater amounts or longer than prescribed and it is important to determine the extent to which overuse occurs, and the reasons underlying it. Indeed, according to a US survey, up to a quarter of ADHD patients reported overusing their prescribed medication and taking higher doses than prescribed [30]. The authors noted that physicians should be vigilant for possible overuse and/or diversion of PS among ADHD patients attending college.

### Patterns of timing and age of first-use

Only a small proportion of misusers used PS at least once a week during the year, while use during internships, parties or sports competitions was even more limited. This pattern of use is similar to other studies conducted in Flanders and in Europe [2, 16]. However, attention has to be paid to a minority of misusers who frequently use PS during exam periods, as this may lead to cardiovascular problems or dependence [14].

In our study, the age of onset of medical use was evenly distributed across age categories, with one-third starting use after the age of 18. According to a meta-analytic review, the overall prevalence of ADHD was higher in preschool and elementary school sample and then decreased in adolescents and adulthood samples [31]. We found that, similarly to the Flanders results, most misusers started after the age of 18 years [16].

### Sources and routes of administration

Friends and acquaintances were frequent sources of PS for misuse. This finding suggests that some medically treated patients transfer their medicines to friends or acquaintances and confirms the possibility of access to PS for misuse, either through the possibility of obtaining a prescription for oneself or through social networks [2, 22].

Over one-fifth of misusers reported obtaining these medications through a GP. A survey among GPs in Belgium concluded that subjective norms and attitudes strongly influence a physician’s intention to prescribe MPH for cognitive enhancement [32]. In particular, GPs who perceived greater social pressure from others in their lives, those with more favourable attitudes, and those who perceived that it is their own choice to prescribe MPH were more likely to show an intention to prescribe MPH to students to improve their academic performance. In France, an increase in doctor shopping behaviour for MPH was observed and estimated to be as high as for other psychoactive drugs like opioids [33]. Moreover, a European Community pharmacy study reported that MPH had the highest number of falsified prescriptions, particularly in France, Spain and Belgium [34]. Health care professionals should be aware of this behaviour and the possibility that patients may feign symptoms to obtain fraudulent prescriptions [35]. One of the recommendations of an expert group in Belgium was that starting and refining medicinal treatment should be done by a (paediatric) psychiatrist and treatment could then be continued under supervision of a GP, with annual check-up by the second-line expert [36]. An interview-based study investigated the social context of GPs’ decisions to prescribe off-label stimulants. Two groups of GPs could be identified: those who strictly follow medical guidelines and those who, depending on the context, prescribe stimulants according to patients’ symptoms and the extent of their needs [37].

In a US survey, only a small proportion of PS misusers (1.8%) reported obtaining the medication through the internet [38]. This is in contrast to our study, where almost one in ten misusers obtained stimulating drugs via the internet. In Belgium, prescription-only medicines can only be delivered in a pharmacy and it is illegal to obtain them online [39]. Moreover, these products do not always contain the right active ingredient, the right dosage or the right excipients.

Route of administration was also questioned as previous reports indicate that misuse of PS is mainly by oral and to a lesser extent trough intranasal administration [40]. However, when non-oral use is reported, this has been associated with an increased likelihood of serious, non-alcohol substance use [41]. While an overwhelming majority of medical users of PS reported oral administration, 10% of misusers reported nasal administration. Although there is little evidence to support this, non-oral routes of administration may increase the risk for serious effects and increase vulnerability to addiction [5, 42].

### Perceived effects

In line with the main reason for using PS, the most reported beneficial effect in our study was improved concentration. This effect was reported more frequently, together with more frequent reports of better academic performance, in medical users. A Cochrane systematic review concluded that in children and adolescents, MPH may improve teacher-reported symptoms of ADHD and general behaviour [43].

Compared to medical users, the positive effects among misusers were more related to the stimulating effect (e.g. staying awake longer). In Swiss university students, 68% of PS users reported that these substances met their expectations with regard to expected effects on neuroenhancement [23]. Previous studies have shown that there does not seem to be any academic advantage or benefit associated with misuse of PS [2, 44].

Palpitations and agitation tended to be reported more frequently in misusers than in medical users, while anorexia was more reported in medical users. This could be due to the dosage or frequency of use among these different type of users.

### Consumption of other substances

In our survey, PS users were more likely to use other commonly misused and abused substances such as nicotine, hypnotics and sedatives. Studies on neuroenhancement have consistently examined the positive relationship between neuroenhancement and risky health behaviours such as recreational drug use [23]. In general, students experienced with neuroenhancement were more likely to engage in problematic legal and illegal drug use [35, 45]. However, these associations can lead to serious psychological, physical, legal or even fatal complications [46]. A survey of college students in the US found that students who perceived a more academic benefit from nonmedical use of PS and who drank alcohol and used cannabis more frequently were more likely to engage in misuse of PS [47]. Adult ADHD often co-occurs with substance use disorders and is associated with reduced effectiveness of standard treatments. Clinicians face a dilemma when prescribing stimulants in this high-risk population, but close monitoring, the use of long-acting formulations and a commitment of patients to safeguard their medication, can reduce misuse and diversion [10].

### Strengths and Limitations

The study size was large and the mode of survey administration was appropriate for the target population (young, highly educated, access to Internet, regular use of e-mail). Moreover, online surveys may also be less affected by social desirability effects for sensitive questions. Furthermore, the questionnaire was distributed through the academic authorities of the universities, which could also have increased the level of confidence in the survey [48].

An accurate response rate could not be calculated because the actual number of contacted subjects was only roughly estimated. Non-response bias is an important potential source of bias in self-administered questionnaires. In the present study, the effect of bias could act in two opposite directions: an upwards bias (i.e., overestimated prevalence) because the most concerned subjects may participate more actively, and downward bias (i.e., underestimated prevalence) because of social desirability effects, confidentiality concerns and under-representation of the male population.

The prevalence of PS use was negatively correlated with the level of completeness of the questionnaire. Subjects who provided partial responses had high levels of PS use. This finding is difficult to evaluate; participants who completed the questionnaire in full may respond, on average, more conscientiously than those who did not answer all the questions. However, it is also possible that a fatigue/boredom effect played a role, especially in subjects with some particular characteristics (e.g., more easily distracted). It is also worth noting that the prevalence of PS use was significantly higher among students who defined their gender as “other”. This could suggest a potential “jokester effect” (i.e. intentionally providing inaccurate responses) [49, 50] or the presence of a true profile specific to this subgroup.

Online surveys are known to select respondents with a particular interest in the topic under study. Previous studies have shown that non-response is usually associated with pathology and that underreporting of problem behaviours is more likely [51]. On the other hand, as participation was voluntary, anonymous, and the survey was web-based, there is less reason to assume that the data collection procedure would lead to socially desirable answers. International students were able to participate to this research, although the survey was only available in French. Students who did not feel comfortable communicating in French were certainly under-represented. This could not be further explored as the questionnaire did not include questions on e.g. country of origin.

## Conclusions

This survey was the first to assess the prevalence of medical use and misuse of PS among students at French-speaking universities in Belgium. The results show that the prevalence of use of PS was around 7%. This prevalence is in line with similar studies conducted in Flanders and Europe.

These findings have to be interpreted with caution given the participation rate and the potential for non-response bias. This study also confirms that the primary motivation for PS misuse is the desire to improve school performance. However, misuse or motivations might change in the context of the COVID-19 pandemic. According to a longitudinal cohort study in adolescents in the US, a decreased use of alcohol and increased use of nicotine and misuse of prescription drugs was noted during the pandemic [52]. Rates of use were higher among financially distressed families and emotionally distressed youth. These findings should be taken into account in future research and potential strategies to mitigate risk of substance use. It may also be beneficial to examine the effectiveness of interventions if and when offered within academic settings.

## Data Availability

The data that support the findings of this study are available from the corresponding author, MS, upon reasonable request.

## Abbreviations

AE: Adverse Events
ADHD: Attention-Deficit/Hyperactivity Disorder
DDD: Defined Daily Dose
GP: General Practitioner
MPH: Methylphenidate
PS: Prescription Stimulants
ULB: Université Libre de Bruxelles
US: United States
